# Pylorus-preserving laparoscopic proximal gastric resection with Roux-en-Y esophagojejunostomy for late gastrocolic fistula after sleeve gastrectomy

**DOI:** 10.1093/jscr/rjag928

**Published:** 2026-10-22

**Authors:** Oktyabr Teshaev, Nozim Jumaev, Ziyoda Muminova, Irina Lim, Jafar Zafarovich Juraev, Azam Bobabekov

**Affiliations:** Department of Surgical Diseases, Tashkent State Medical University, Tashkent, Uzbekistan; Department of Surgical Diseases, Tashkent State Medical University, Tashkent, Uzbekistan; Department of Obstetrics and Gynecology, Reproductology, Tashkent State Medical University, Tashkent, Uzbekistan; Department of Surgical Diseases, Tashkent State Medical University, Tashkent, Uzbekistan; Department of Surgical Diseases, Tashkent State Medical University, Tashkent, Uzbekistan; Department of Surgical Diseases, Tashkent State Medical University, Tashkent, Uzbekistan

**Keywords:** sleeve gastrectomy, staple-line leak, gastrocolic fistula, laparoscopic revision, Roux-en-Y reconstruction, case report

## Abstract

Laparoscopic sleeve gastrectomy (LSG) is among the most common metabolic bariatric procedures, but staple-line leak occurs in 1%–3% of cases and may evolve into chronic fistula. Gastrocolic fistula is rare and lacks standardized treatment. We describe a 44-year-old woman (body mass index 43 kg/m^2^) who developed a late staple-line leak 7 months after LSG that progressed to a gastrocolic fistula, with mega-stent-related pressure necrosis of the transverse colon as the proximate mechanism. After failed conservative and endoscopic management, laparoscopic revision was performed: adhesiolysis, proximal gastric resection, primary repair of the 4-cm colonic defect with omental patch, and Roux-en-Y esophagojejunostomy with anterior cruroplasty. The patient was discharged on postoperative day 15 and remained asymptomatic through 12 months, with intact anastomoses and no recurrence. Unlike previously reported proximal, near-total, or total gastrectomies, the distal antrum and pylorus were preserved. Laparoscopic revision is feasible and effective for this complex complication after LSG.

## Introduction

Laparoscopic sleeve gastrectomy (LSG) is one of the most frequently performed metabolic and bariatric procedures worldwide, owing to its technical simplicity and metabolic efficacy [1]. Staple-line leak remains among its most serious complications, occurring in 1%–3% of cases [2], and late presentations may be atypical and progress to complex fistulae [3]. Gastrocolic fistula after LSG is rare: a recent systematic review identified only 13 case reports and three retrospective cohorts across the metabolic bariatric surgery literature, with LSG accounting for most cases and the transverse colon the most frequent site [4]. Its pathogenesis involves wall ischaemia, infection of a hematoma or abscess, and stent or drain erosion [5]. Management is multidisciplinary and non-standardized, ranging from conservative and endoscopic therapy to surgery of varying extent [6]. We describe laparoscopic management of a late gastrocolic fistula after LSG by proximal gastric resection with Roux-en-Y esophagojejunostomy. This report follows the Surgical CAse REport (SCARE) 2023 guideline [7].

## Case report

A 44-year-old woman with clinically severe obesity (height 160 cm, weight 110 kg, body mass index 43 kg/m^2^) underwent LSG at another institution; operative details of the index procedure were unavailable.

Seven months postoperatively she presented with dizziness, weakness, and severe vomiting. Esophagogastroduodenoscopy (EGD) before any stent showed a fistulous tract at the staple line at the esophagogastric junction through which a purulent cavity drained into the gastric lumen (Fig. 1)—an initially contained staple-line leak walled off as a large peri-gastric abscess. A covered mega-stent was placed across the gastric tube; the normal white-cell count despite the abscess reflected the chronic, walled-off process.

**Figure 1:**
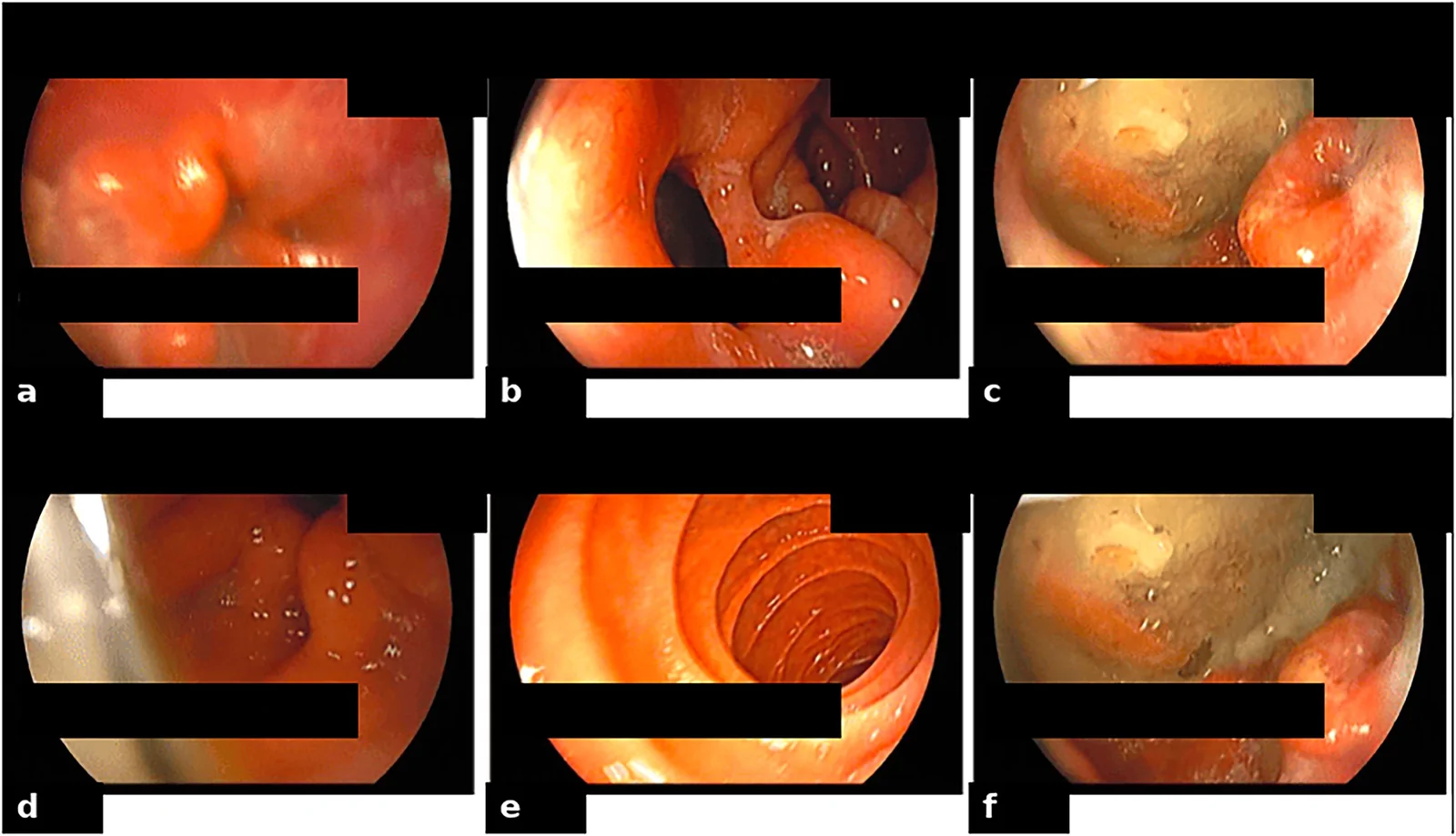
(a–f) Endoscopic findings before stent placement. Esophagogastroduodenoscopy at the referring institution 7 months after LSG, prior to stenting, showing a fistulous tract at the staple line at the esophagogastric junction with chronic inflammatory change, through which a purulent cavity communicated with the gastric lumen - an initially contained staple-line leak walled off as a large peri-gastric abscess.

After 6 weeks the stent was removed without improvement, and she was referred to us with constant epigastric and left upper-quadrant pain, low-grade fever (37.2°C–37.5°C), weight loss, and feculent vomiting. Laboratory studies showed anemia (haemoglobin 88 g/L), hypoproteinemia (52.5 g/L), hypoalbuminemia (25.5 g/L), and elevated erythrocyte sedimentation rate (ESR) and C-reactive protein (CRP) with a normal white-cell count (8.48 × 10^9^/L). Repeat EGD identified two defects—the purulent staple-line cavity and a new gastrocolic fistula transmitting fecal content into the stomach—attributed to sustained mega-stent pressure with necrosis of the adjacent transverse colon. Contrast radiography confirmed extravasation from the gastric lumen into the abscess cavity and communication with the transverse colon (Fig. 2). The clinical course is summarized in Table 1.

**Figure 2:**
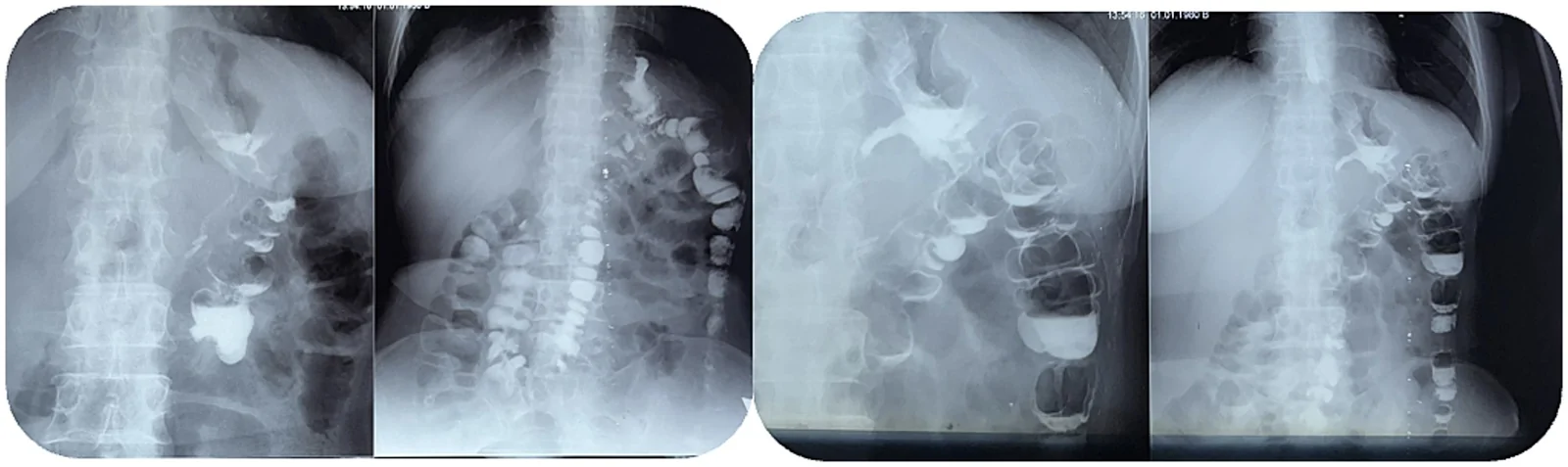
Contrast radiography confirming the gastrocolic fistula. (a) Anteroposterior view: Contrast extravasation from the gastric lumen into the perigastric abscess cavity. (b) Lateral view: Contrast passage into the transverse colon lumen.

**Table 1:** Clinical timeline of events.

**Timepoint**	**Event**	**Management / findings**
Day 0	Primary LSG (outside institution)	BMI 43 kg/m^2^; uneventful early course.
Month 7	Symptom deterioration; EGD (pre-stent)	Staple-line fistula at the esophagogastric junction; contained peri-gastric abscess; covered mega-stent (24 mm) placed.
Month 8.5	Stent removal; referral; repeat EGD	No improvement; feculent vomiting; two defects - staple-line cavity and new gastrocolic fistula from stent pressure necrosis.
POD 0	Laparoscopic revision (180 min)	Adhesiolysis; proximal gastric resection; colonic repair with omental patch; Roux-en-Y esophagojejunostomy; cruroplasty.
POD 15	Discharge	Uneventful postoperative course.
12 months	Long-term follow-up	Asymptomatic; anastomoses intact; no recurrence.

Abbreviations: BMI, body mass index; EGD, esophagogastroduodenoscopy; LSG, laparoscopic sleeve gastrectomy; POD, postoperative day

After 3-day multidisciplinary optimization (nutritional support, correction of anaemia and electrolytes, and formal risk assessment), laparoscopic revision was undertaken via a five-port technique. Dense upper-abdominal adhesions, a fibrotic omentum, and an extensive purulent cavity adjacent to the liver, spleen, and pancreas were found. After adhesiolysis, the fistulous tract between the proximal sleeve and transverse colon was divided and the cavity evacuated (Fig. 3). A 5-cm gastric staple-line defect with dense fibrosis precluded primary repair; proximal gastric resection to healthy margins was performed, preserving the distal antrum and pylorus (Fig. 4). The 4-cm colonic defect was closed with single-layer non-absorbable sutures and an omental patch. As the short, densely adherent gastric remnant could not reach the esophagus without tension, continuity was restored by Roux-en-Y esophagojejunostomy; the preserved antrum, pylorus, and duodenum were left as the biliopancreatic limb, draining through a jejunojejunostomy 50 cm distal to the esophagojejunostomy. Anterior cruroplasty was added for antireflux protection (Fig. 5). Operative time was 180 minutes with minimal blood loss.

**Figure 3:**
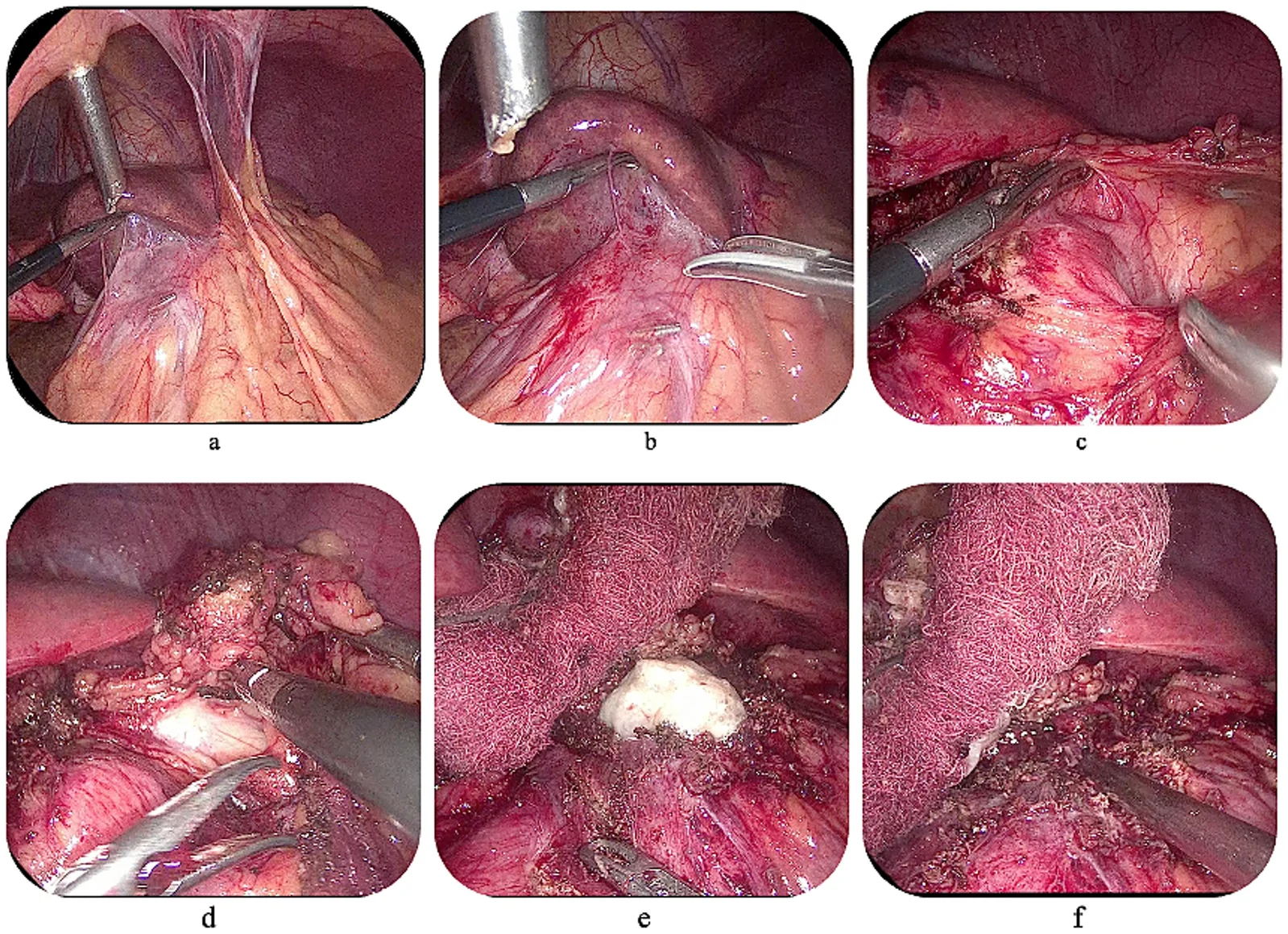
(a–f) Adhesiolysis and fistula exploration. Dense upper-abdominal adhesions, dissection of the inflammatory conglomerate with identification of the fistulous tract between the proximal gastric sleeve and the transverse colon, and evacuation of the chronic purulent cavity adjacent to the liver, spleen, and pancreas.

**Figure 4:**
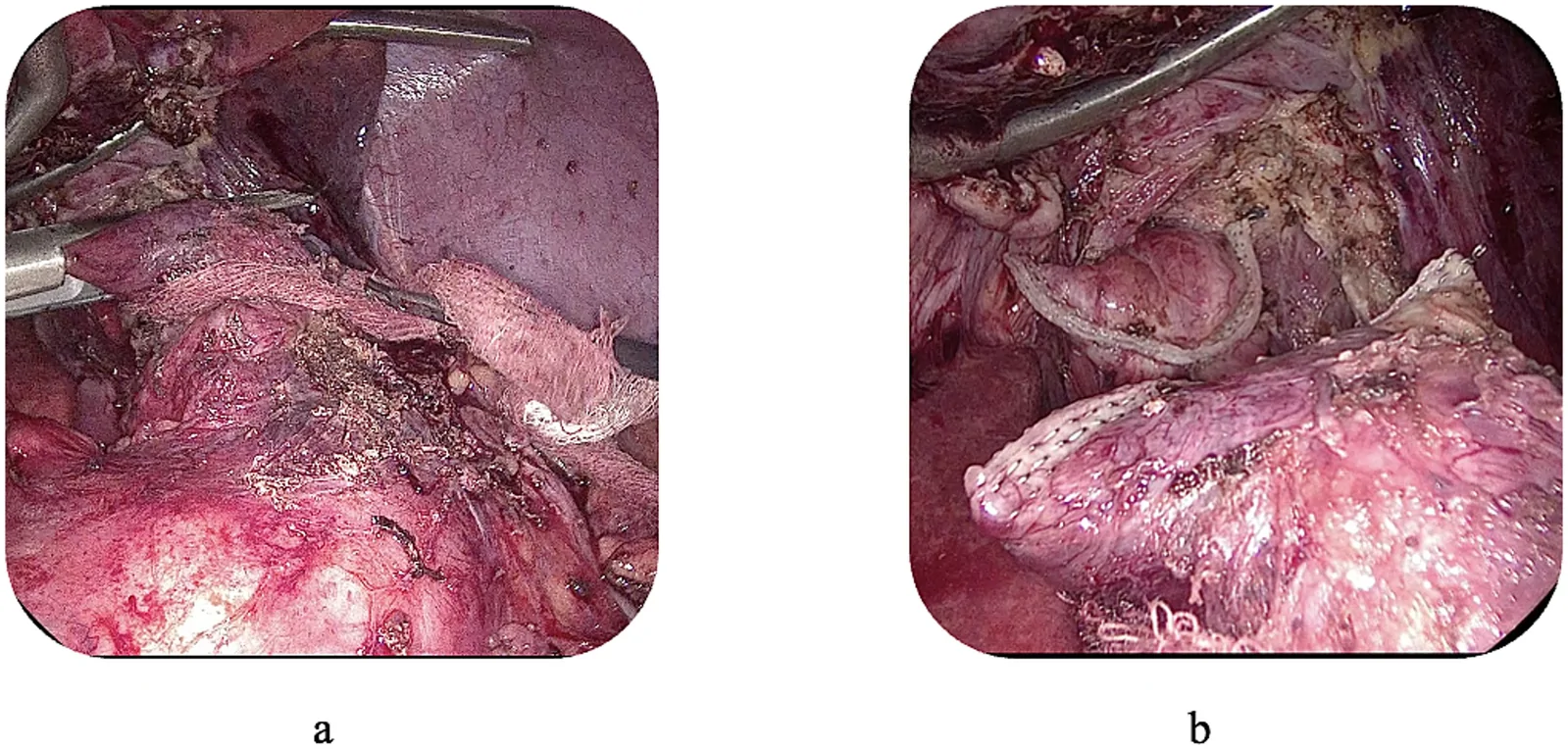
Proximal gastric resection (a, b). Mobilization of the proximal sleeve segment bearing the staple-line defect with vascular ligation, and resection to healthy margins with preservation of the distal antrum and pylorus.

**Figure 5:**
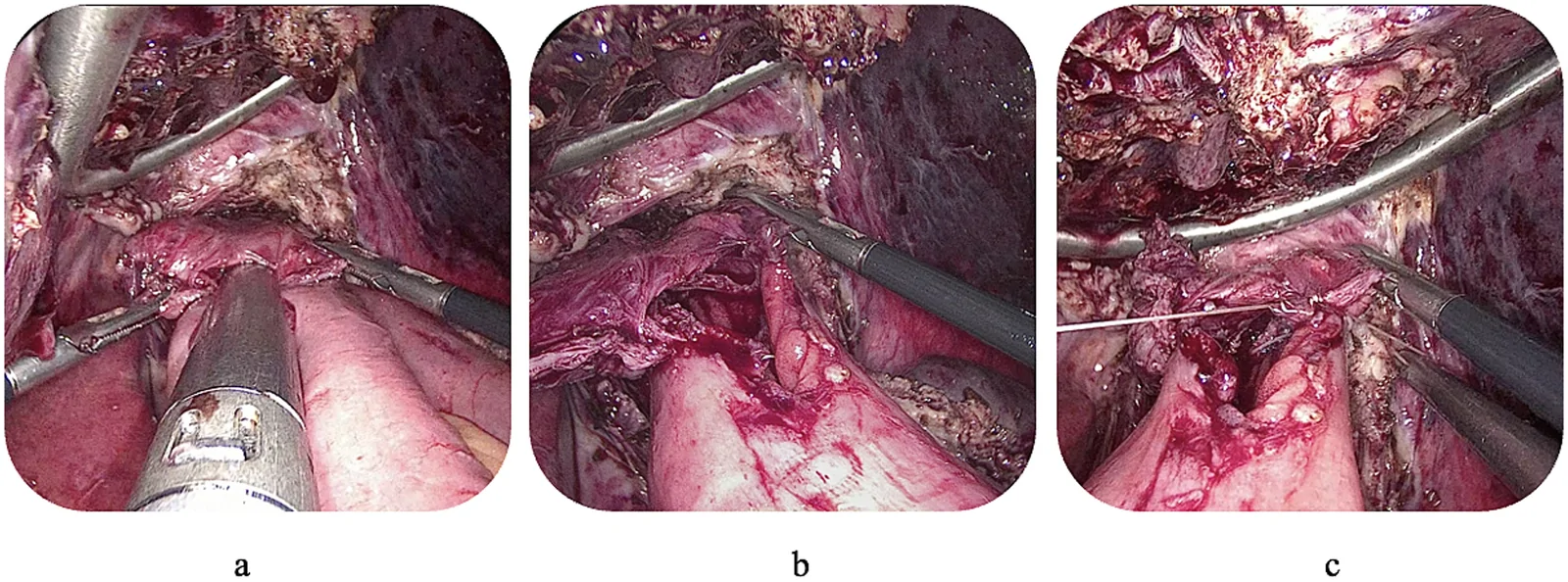
(a–c) Reconstruction and antireflux protection. Roux-en-Y reconstruction with end-to-side esophagojejunostomy, and anterior cruroplasty with approximation of the diaphragmatic crura using non-absorbable sutures.

A water-soluble contrast study on postoperative day (POD) 2 confirmed anastomotic integrity, oral intake was resumed, drains were removed on POD 3, and antibiotics were continued for 12 days. Through 12 months of follow-up she remained asymptomatic, and control imaging and endoscopy showed patent, non-stenotic anastomoses without reflux or recurrence.

## Discussion

This case illustrates a rare complication of LSG—late staple-line failure with gastrocolic fistula - notable for its late onset and aggravation by stenting. By the Csendes classification (early, Days 1–4; intermediate, 5–9; late, ≥10 days), this leak arose far beyond the late window and behaved as a chronic, walled-off failure [3].

The mega-stent, placed to bridge the leak and associated narrowing, paradoxically completed the fistula: sustained pressure on inflamed tissue adjacent to the colon produced necrosis [8]. This underscores caution with stenting when a chronic contained leak involves adjacent viscera. Endoscopic treatment succeeds in many LSG leaks but far less often in late, complex, fibrotic fistulae [9], as here. Attention to modifiable technical factors at the index operation—staple-line reinforcement and bougie calibration—remains central to prevention [10, 11].

Given the defect sizes (gastric 5 cm, colonic 4 cm), adjacent involvement, and failed conservative therapy, laparoscopic revision with resection and Roux-en-Y reconstruction was selected; despite dense adhesions the laparoscopic approach was feasible [12] and reduced operative trauma. Two features distinguish this case. First, the distal antrum and pylorus were preserved, whereas previously reported resections were proximal, near-total, or total gastrectomies [5, 13, 14]. Anatomic preservation avoids total gastrectomy and retains reconstructive options, with the pylorus continuing to regulate biliopancreatic outflow through the duodenum while food passes through the esophagojejunostomy. Second, the stent placed to bridge the leak was directly implicated in completing the fistula, consistent with reservations about stenting raised in the systematic review [4].

This single case may not be generalizable, and because the index procedure and stenting were performed elsewhere, their technical details were unavailable; longer follow-up is needed to confirm durability and nutritional outcomes. Nonetheless, late gastrocolic fistula after LSG is rare but serious, and laparoscopic pylorus-preserving proximal gastric resection with Roux-en-Y esophagojejunostomy is feasible and effective even with dense adhesions in experienced centers.

## Data Availability

The data supporting this case report are available from the corresponding author on reasonable request.

## Image integrity

The endoscopic and radiological images were modified solely to remove patient- and institution-identifying information (de-identification). No image content was fabricated, enhanced, obscured, or otherwise manipulated in any way that could affect scientific interpretation.
